# Risk of birth defects in children conceived by artificial oocyte activation and intracytoplasmic sperm injection: a meta-analysis

**DOI:** 10.1186/s12958-020-00680-2

**Published:** 2020-12-11

**Authors:** Rui Long, Meng Wang, Qi Yu Yang, Shi Qiao Hu, Li Xia Zhu, Lei Jin

**Affiliations:** Reproductive Medicine Center, Tongji Hospital, Tongji Medical College, Huazhong University of Science and Technology, 1095 Jiefang Ave, Wuhan, 430030 China

**Keywords:** Birth defects, AOA, ICSI, Meta-analysis, Chromosomal aberrations

## Abstract

**Background:**

Whether artificial oocyte activation (ICSI-AOA) will increase the risk of birth defects remains controversial. Thus, we performed this study to evaluate the risk of birth defects and further compare the incidence of different birth defects types (chromosomal aberrations and non-chromosomal aberrations) in children conceived by ICSI-AOA and conventional intracytoplasmic sperm injection (ICSI) in an enlarged sample size.

**Method:**

A comprehensive review of the literatures comparing birth defects in children conceived by ICSI-AOA and conventional ICSI by October 2020 was performed in PubMed, Embase, Cochrane Libraries, Web of Science, and Chinese databases including China National Knowledge Infrastructure, China Biology Medicine disc and Wan Fang. Risk ratios (RR) and 95% confidence intervals (CI) were calculated.

**Results:**

Five studies were included in the final analysis. Compared with conventional ICSI, ICSI-AOA did not increase the birth defects rate (RR = 1.27, 95%CI 0.70–2.28) of children. Furthermore, in a subgroup analysis, birth defects were classified into two types (chromosomal aberrations and non-chromosomal aberrations) in four studies and no statistical difference were revealed.

**Conclusion:**

Our analysis indicates that ICSI-AOA represents no significant difference in the prevalence of major birth defects or types of birth defects (chromosomal aberrations and non-chromosomal aberrations) comparing with conventional ICSI. This conclusion may provide clinicians evidence-based support in patient counseling and instruction of the application and safety concern about ICSI-AOA.

## Background

Since the first intracytoplasmic sperm injection (ICSI) pregnancy were reported in the early 1990s [1], ICSI has been used as an effective method for assisted reproductive technology (ART), especially for patients with suboptimal semen parameters or zero or low fertilization rates after conventional in vitro fertilization (IVF). However, ICSI results in average fertilization rates of 70% [2], total fertilization failure (TFF) still occurs in 1–3% cycles and can recur in subsequent cycles, even when a sufficient number of oocytes and motile spermatozoa are available [3, 4].

Oocyte activation which induces the calcium oscillations to raise the intracellular calcium levels in the oocyte after spermatozoon-oocyte fusion [5], is a master key to initiate all the cytological changes in fertilized oocyte [6, 7]. Therefore, oocyte activation failure is generally regarded as the principal cause of TFF [4, 8, 9].

Artificial oocyte activation (AOA) is considered as an effective method to improve fertilization rate after TFF [10]. A variety of activating oocyte methods have been applied in human assisted reproduction treatment, including physical, mechanical or chemical stimuli, mainly aiming to initiate artificial Ca^2+^ rises in the oocyte cytoplasm [11]. Previous study has reported that the fertilization rate increased from 25 to 48% after using AOA [10]. And the applications of these methods in clinical have been previously reviewed [12–14].

However, AOA procedure, which includes an additional manipulation on the injected oocyte, and incubation in activating agents, may interfere the cell metabolism or embryo development. As previously reported, gamete manipulations, which were both involved in AOA and conventional ICSI procedures were considered to be possible risk factors in birth defects [15]. The calcium rises induced artificially are not able to mimic calcium oscillations in physiological conditions precisely and little has been known yet about the possible side effects of ionophores on post-implantation embryo development [14]. Therefore, there is always a concern whether the children conceived by ICSI-AOA have a poor neonatal outcome. Previous studies have demonstrated that there are no significant differences in gestational week, birth weight, preterm birth rate or gender ratio between conventional ICSI and ICSI-AOA [15–19]. However, regarding the aspect of birth defects, some publications have reported increased risks in infants born after ICSI-AOA compared with those after conventional ICSI [19, 20]. Whereas, some studies held the opposite views that ICSI-AOA did not affect the incidence rate of birth defects [10, 17, 18, 21, 22]. In addition, the prevalence of chromosomal anomalies has been reported to be higher in conventional ICSI pregnancies [23, 24]. Considering that AOA takes place during the time of meiotic spindle orientation and completion of meiosis [25], there is probably a higher chromosomal aberrations (CA) risk to children conceived by ICSI-AOA. Therefore, it is also necessary to assess the risk of birth defects type, chromosomal aberrations (CA) and non-chromosomal aberrations (NCA), respectively.

Due to the indications and the unknown safety problems, ICSI-AOA is not considered as a routine practice of ART yet, which is only suitable for patients with proper indications, including low or zero fertilization in the previous ICSI cycles or poor quality of sperm. The number of babies born from infertile patients undergoing ICSI-AOA was limited, so that the sample size of current studies was too small to draw a concluded outcome, especially in a single center. This meta-analysis aimed to enlarge born babies sample size to address the safety of AOA by comparing the risk of birth defects in children conceived by ICSI-AOA and conventional ICSI and assess the risk of birth defects type (CA and NCA).

## Materials and methods

The proposed PRISMA (Preferred Reporting Items for Systematic Reviews and Meta-Analyses) guidelines were followed to report the present review [26].

### Search strategy

**Fig. 1:**
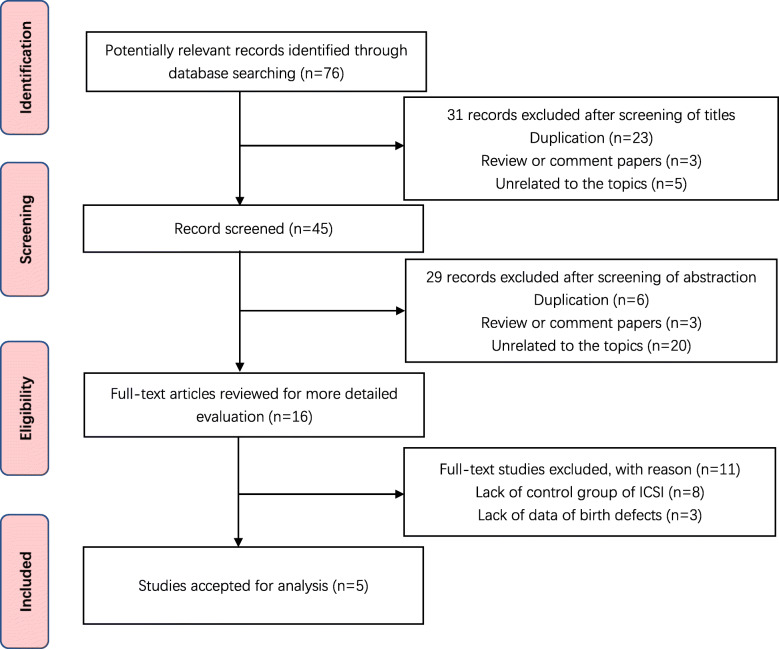
A PRISMA statement flow diagram

We aimed to identify all relevant studies that compare the outcome of children conceived by ICSI-AOA and conventional ICSI. A systematic literature search was conducted of PubMed, Embase, Cochrane Libraries, Web of Science, and Chinese databases including China National Knowledge Infrastructure (CNKI), China Biology Medicine disc (CBM) and Wan Fang. All publications appearing before October 2020 in these databases were included. The following terms were used, adjusting for each database as necessary: (((Calcium ionophore) AND ((Intracytoplasmic sperm injection) OR ICSI)) AND Oocyte activation) AND ((((Congenital abnormalities) OR Birth defects) OR malformations) OR follow up). Additionally, we hand-searched the reference list from included trials and similar reviews, and all citations identified were reviewed, irrespective of language. Two independent investigations reviewed study titles and abstracts, and studies that satisfied the inclusion criteria were retrieved for full-text assessment. The search strategy and included studies are shown in Fig. 1.

### Selection criteria

Studies were included if 1) the exposure of interest was ICSI-AOA or conventional ICSI; 2) the outcome of interest was birth defects; 3) comparison of ICSI-AOA to conventional ICSI; 4) risk ratio (RR) with 95% confidence intervals (CI) provided or could be calculated; 5) the study was published in English or Chinese. The exclusion standard was that 1) studies without full data; 2) studies were case reports or reviews; 3) studies with inappropriate comparison group or without control subjects.

### Data extraction

Two reviewers independently reviewed all the articles, and identified the information from each study blindly. Quantitative data were collected as follows: authors, publication year, geographic region, study design, sample size of ICSI-AOA and conventional ICSI, the number and the system of birth defects and adjustment for confounders, and other related information. Study authors were contacted for the details to ensure accuracy in the review. The data were checked by other investigators. Disagreement between reviewers regarding data abstraction were resolved through discussion. Two independent reviewers assessed risk for bias according to the PRISMA recommendations [26].

### Assessment of study quality

Newcastle–Ottawa Scale which has eight items was used to assess the quality of the studies [27]. One or two points were awarded for each criterion and the points were added up to compare study quality in a quantitative manner. Total points of <7 and ≥ 7 were assigned for low and high quality of studies, respectively. Two reviewers carried out the assessment independently. Any disagreements were resolved through discussion until consensus was reached.

### Data synthesis

Due to the rare number of the birth defects, we assumed equivalence of the odds ratio and RR, and regarded RR as an effective measure of the relationship between AOA/ICSI and risk of birth defects and chromosomal aberrations across studies. Statistical heterogeneity among studies was evaluated by using the Q statistic (significance level at *P* <0.1), and the I^2^ statistic (significance level at I^2^>50%). A fix-effected model was used to calculated RR and 95%CI if the homogeneous test was not significant (*P* >0.1). Otherwise, a random-effects model was used. Publication bias was assessed by funnel plots and the Begg’s test (significance level at *P* <0.1). All statistical tests were calculated using the Review Manager 5.1 software.

## Result

### Study inclusion and basic

Through database searching, 76 potential eligible records were found. Of these, 31 were not relevant and 29 were duplicates. Full-text reading of remained 16 studies was retrieved for review, 10 were journal articles whereas 6 were conference abstracts. Eleven studies were excluded because 8 trials didn’t compare AOA to ICSI and 3 trials didn’t involve the data of birth defects. Finally, 5 studies were included [15–18, 28]. Reviewers had perfect agreement in selecting these 5 studies using the stated eligibility criteria.

### Study characteristics

Characteristics of included studies, published between 2013 and 2019, which involved 5506 conventional ICSI infants and 316 ICSI-AOA infants, are summarized in Table 1. The sample size of conventional ICSI and ICSI-AOA in each study ranged from 89 to 2442 and 18–95, respectively. Furthermore, 80% of the studies stratified birth defects to CA and NCA.

**Table 1:** Characteristics of included studies of birth defects in babies from conventional ICSI and ICSI-AOA pregnancies

Author(s) Publication year	Location	Study design	Time-period	Length of follow-up	Included study population	Methods of oocyte activation	Conventional-ICSI		ICSI-AOA	
							No. of children (singletons/multiples)^**a**^	Children with birth defects	No. of children (singletons/multiples)^**a**^	Children with birth defects
Deemeh (2015) [18]	Iran	Historical cohort study	2008–2010	1–30 months	Live births	ionomycin	89 (67/22)	2 (2.2%)	79 (68/2019)	0 (0%)
Nakajo (2016) [16]	Japan	Retrospective cohort study	1995–2014	6 years	Live births	Ca^2+^ ionophore/SrCl_2_	1978 (1640/338)	75 (3.8%)	62 (51/11)	2 (3.2%)
Miller (2016) [15]	Israel	Retrospective cohort study	2006–2014	Birth	Live births and TOP	Ca^2+^ ionophore	426 (315/111)	26 (6.1%)	62 (51/11)	6 (9.7%)
Li B (2019) [17]	China	Retrospective cohort study	2011–2016	Birth	Live births	ionomycin	2442 (1504/938)	31 (1.3%)	95 (59/36)	2 (2.1%)
Kobayashi (2013) [28]	Japan	Retrospective cohort study	2006–2012	Not known	Live births	ionomycin	571 (Not known)	8 (1.4%)	18 (Not known)	1 (5.5%)
Total							5506	142 (2.6%)	316	11 (3.5%)

*ICSI* intracytoplasmic sperm injection, *AOA* artificial oocyte activation, *TOP* terminal of pregnancy

^a^Multiples including twins and triplets

All the included studies were retrospective in nature and activated oocyte by chemical stimuli, including ionomycin [17, 18, 28], Ca^2+^ ionophore [15, 16] or SrCl_2_ [16], which was summarized in Table 1. Due to the lack of a uniform standard, different indications of AOA were used in the included studies. Deemeh et al. [18] performed ICSI-AOA with previous failed or low (< 40%) fertilization rate and 100% sperm abnormality of different types. Miller et al. [15] offered the AOA procedure for patients who had failed fertilization after one ICSI procedure in the presence of at least five mature oocytes without oocyte abnormality or had < 10% fertilization rate. In the study of Bin Li et al. [17], patients with ICSI-AOA should meet one of the following criteria: ≤50% ICSI fertilization rate; good quality embryo rate ≤ 30%; the presence of severe oligoasthemoteratozoospermia; surgically retrieved sperms from testicular sperm aspiration (TESA) or percutaneous epididymal sperm aspiration (PESA). The other two studies [16, 28] were not mentioned their indications.

Three of them [15, 17, 18] only included major birth defects and showed the specific organ system involvement which are summarized in Table 2. Data about birth defects were collected from pediatric report or questionnaires, and then reviewed by pediatrician or board-certified medical geneticist. The remained studies [16, 28] did not mention these details about birth defects. Some studies [16, 18] monitored the occurrence of birth defect in the offspring at least 1 year after birth. Another two studies diagnosed birth defects only at birth [15, 17]. And one study [28] did not mention about the follow-up time. Nakajo et al. [16] divided the conventional ICSI into three sub-groups, ejaculated sperm ICSI group (ej-ICSI), testicular sperm extraction group (TESE) and in vitro maturation group (IVM). It is well known that testicular sperm have higher rates of aneuploidies and diploidy, which means a higher incidence of congenital defect [29]. Therefore, we excluded the cycles used testicular sperm to avoid any bias in the results. The other studies assessed the birth defects in conventional ICSI and ICSI-AOA, respectively.

**Table 2:** Number of birth defects in specific organ system from included studies

Author(s)	Specific organ system	Circulatory system	Genitourinary system	Musculoskeletal system	Digestive system	Nervous system	Face	Beckwith-Weidemann syndrome	Poland syndrome
**Deemeh** [18]	**AOA**	0	0	0	0	0	0	0	0
	**ICSI**	1	0	1	0	0	0	0	0
**Miller** [15]	**AOA**^**a**^	4	2	2	0	0	0	1	0
	**ICSI**^**b**^	8	5	3	2	3	1	0	0
**Li B** [17]	**AOA**	2	0	0	0	0	0	0	0
	**ICSI**	20	1	3	0	0	3	0	1

*ICSI* intracytoplasmic sperm injection, *AOA* artificial oocyte activation

^a^In the AOA group, one case with several structural defects (dysplastic kidney, reflux, ventricular septal defect) and another case with ventricular septal defect, interrupted inferior vena cava and short thumb with low insertion of right hand

^b^In the ICSI group, one case with both hypospadias and ventricular septal defect

Four studies [16–18, 28] only included the birth defects of live birth and one study [15] included live birth and terminations of pregnancy for fetal anomaly. Consanguineous marriages of first-cousin union were included in one study [18] and the consanguineous marriages rate was 29.1%, within the reported ranges of first-cousin union previously reported in that country. Total number of conventional ICSI and ICSI-AOA involved in birth defects types is summarized in Table 3.

**Table 3:** Number of children with types of birth defects from included studies

Author(s)	Total number of children		Non-chromosomal aberrations		Chromosomal aberrations	
	ICSI	AOA	ICSI	AOA	ICSI	AOA
Deemeh [18]	89	79	2	0	0	0
Nakajo [16]	1978	62	67	1	8	1
Miller [15]	426	62	19	6	7	0
Li B [17]	2442	95	30	2	1	0

*ICSI* intracytoplasmic sperm injection, *AOA* artificial oocyte activation

### Meta analysis

**Fig. 2:**
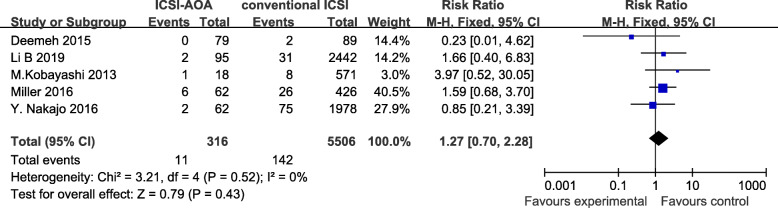
comparison of birth defects rate between children conceived by ICSI-AOA and conventional ICSI (fix effects model). ICSI = intracytoplasmic sperm injection, AOA = artificial oocyte activation

Overall, Fig. 2 showed that there was no significant difference between conventional ICSI and ICSI-AOA group in terms of birth defects risk (RR = 1.27, 95%CI 0.70–2.28, *p* = 0.43). The individual risk estimates for these studies ranged from 0.23–3.97. We found no evidence of heterogeneity of risk ratio among these five studies (*P* = 0.52). Publication bias was not assessed, since the funnel plot analysis was not performed due to the limited study numbers. Sensitivity analysis were performed to assess the influence of each included study on the pooled risk estimate by repeating the meta-analysis after omitting each study in turn. The results suggested that the combined RR was not dominated by any single study.

**Fig. 3:**
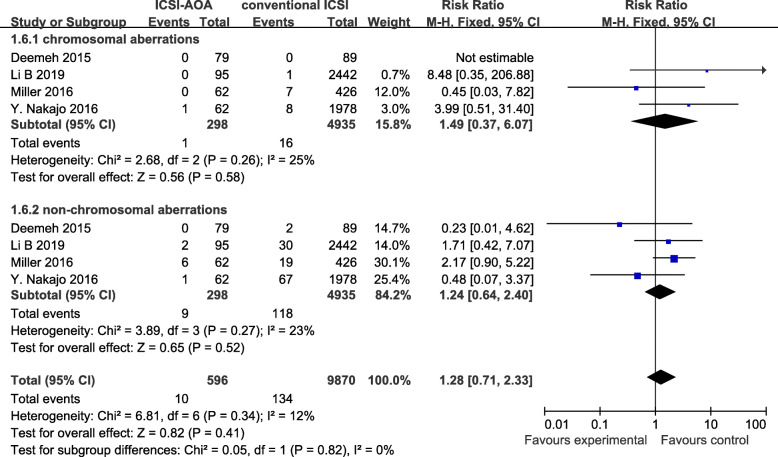
comparison of birth defects type (CA and NCA) between children conceived by ICSI-AOA and conventional ICSI (fix effects model). ICSI = intracytoplasmic sperm injection, AOA = artificial oocyte activation, CA = chromosomal aberrations, NCA = non-chromosomal aberrations

The procedure of AOA mainly affect the time of meiotic spindle orientation and completion of meiosis [25]. Therefore, a subgroup analysis was performed based on the birth defects types (CA or NCA). Figure 3 depicted the results from the fix-effects model combining the RR for CA and NCA. Overall, there were not evidently increased risks for CA (RR = 1.49; 95%CI: 0.37–6.07) and NCA (RR = 1.24; 95%CI: 0.64–2.40). Substantial heterogeneity was not found in these outcomes (all I^2^ ≤ 30%).

## Discussion

### Main findings

We performed a standard meta-analysis to estimate the risk of birth defects in children conceived by ICSI-AOA and conventional ICSI. This meta-analysis included a total of 5506 conventional ICSI infants and 316 ICSI-AOA infants spread over 5 studies. Findings from the present study indicated that there is no significant difference in birth defects between conventional ICSI and ICSI-AOA. According to the type of birth defects, we divided birth defects into CA and NCA. The difference in the prevalence of defects types was not significant. What’s more, substantial heterogeneity was not observed in all outcomes.

### Interpretation

ART is generally considered as a safe method for infertile couples. However, the rapid progress of technologies, as well as the ever-growing demand for ART, make it more important to continually monitor its safety [30]. Since the first report of AOA in 1997 [31], multiple studies have reported increased fertilization and pregnancy rate with the application of AOA as an effective treatment for failed or low fertilization after conventional ICSI [32–34]. Subsequently, the safety of ICSI-AOA has also gained great attention because of the additional manipulation to both oocytes and sperms.

Previous studies have evaluated the bio-safety of ionomycin, strontium chloride and other AOA agents in mice, and no adverse effects were shown on development of murine embryos and health of the filial generation [35, 36]. However, there were no similar studies carried out in human beings. In some retrospective studies which followed up 38, 32, 25, 22, 16, 10 babies from AOA, respectively, no birth defects were reported [21, 22, 37–40]. Nevertheless, Ileana Mateizel et al. reported a cohort of 47 babies delivered after AOA (31 singles and 16 twins) that 3 children were diagnosed with major malformations [19]. Total major malformation rate was 6.3% which was higher than that as described previously in a cohort of children born after using ejaculated sperm ICSI in the same center (4.1%) [41], though it was not significantly different. Another study followed 21 children who were born after AOA for 3–10 years found that they all laid within expected ranges regarding neurodevelopmental, intelligence, language and communication skills, however, 3 children (14.3%) were diagnosed with birth defects at toddler age [20].So, although these two studies were not included in our meta-analysis due to lack of comparative data to conventional ICSI, the application of AOA still should be cautious and the safety of AOA needs to be concerned. Whereas, given the small sample size, these results need to be interpreted prudently.

In this meta-analysis, no sufficient evidence of an increased risk of major birth defects and malformation types after ICSI-AOA as compared with conventional ICSI was found which may imply that AOA does not appear to impose a higher risk of abnormality and AOA may be considered as a safe method for clinical application in ART. The risk of birth defects may be related to the ICSI procedures or an underlying male/female factor rather than AOA. A meta-analysis conducted on 46 studies revealed that children conceived by either IVF and/or ICSI are at a significantly increased risk for birth defects [42]. Sutcliffe AG et al. showed that there were significantly more birth defects among children born from fathers with oligozoospermia than in other children conceived by ICSI in a UK case-control study [43]. Other reports also suggested that the risk of passing on genetic defects to the offspring is highest in men with oligozoospermia [44, 45]. As for female aspects, the incidence of birth defects may associate with maternal age, infertility factors and drugs to stimulate follicular development and ovulation [46, 47]. Furthermore, most of the included studies didn’t include the terminal of pregnancy due to fetal defect, only evaluated the incidence of birth defects in live-born infants, which may lead to underestimating the prevalence of birth defects.

It is interesting that the birth defects after ICSI-AOA and conventional ICSI in our study both had an apparently high proportion of congenital heart defects and genitourinary defects, although numbers were too small for statistical comparisons. Additional larger studies need to be conducted to assess the effect of extra manipulation on embryo and fetal development.

### Strengths and limitation

So far, no meta-analyses have been conducted to explore the association between the AOA infants and birth defects and the possible effect of AOA on the development of embryo chromosome. The present study, to the best of our knowledge, was the first meta-analysis to address these issues. Although only five researches were included, this meta-analysis was really needed in order to pre-estimate the risk of AOA with the increasing concerns on the safety of AOA. An improved understanding of these issues may be beneficial for the development and application of AOA and provide clinicians evidence-based support in patient counseling in assisted reproductive technology.

A study like this will inevitably reveal certain limitations. Firstly, due to its specific indication, the number of patients that may benefit from AOA is rather small, leading to a low number of children born after transfer of an embryo obtained after oocyte activation [19]. So, the association between the risk of birth defects and AOA was limited. As a result, we did not evaluate the safety of the offspring through other subgroup analyses, such as AOA indications, malformed organ systems, oocyte activation methods, fresh or frozen embryo transfer and so on. For such a solid conclusion, more follow-up studies on children conceived by AOA were necessary to further confirm its safety. Secondly, birth defects were only diagnosed at birth in most of the studies [16–18, 28] and a systemic karyotype evaluation was not performed. Some chromosomal anomalies or minor defects, such as certain sex chromosomal anomalies and balanced structural anomalies may go under detected or only be detected in later life. Previous study has reported that the prevalence of these chromosomal anomalies was higher in ICSI pregnancies compared with IVF [42]. Therefore, we cannot exclude a possibility that children conceived by AOA have an elevated prevalence of chromosomal anomalies. In addition, only one included study [16] conducted a six-year follow-up, and examined the cognitive development of children conceived by AOA. So, our study lacked the subgroup analysis about the mental development in AOA infants, which can evaluate the safety of AOA more comprehensively. Thirdly, ideally all pregnancies, also miscarriages and induced abortions due to congenital malformations or chromosomal abnormalities, should be included in the congenital malformation rate. Moreover, in comparison with singletons, twins have higher risks of adverse outcomes including preterm birth, low birth weight and birth defects [48, 49]. Therefore, it is essential to analyze the birth defects rate in singletons and twins, respectively. Whereas, most included studies lacked detailed information about birth defects in these aspects, which was not able to find the association between birth defects and these potential risk factors.

Therefore, large-scale and long-term follow-up studies on the prevalence of AOA-associated birth defects and defects types need to be addressed to support our findings continually in the future. In addition, potential risks of AOA such as mutagenic and epigenetic effects on oocytes and embryos should also be investigated further.

## Conclusion

In conclusion, we did not find a significantly increased risk in terms of birth defects and malformation types in children conceived by ICSI-AOA compared with conventional ICSI in an enlarged sample size analysis. There are still concerns related to the safety of ICSI-AOA and more well-conducted observational studies are needed.

## Data Availability

The current study was based on results of relevant published studies.

## Abbreviations

ICSI-AOA: Artificial oocyte activation
ICSI: Intracytoplasmic sperm injection
RR: Risk ratios
CI: Confidence intervals
ART: Assisted reproductive technology
IVF: In vitro fertilization
TFF: Total fertilization failure
CA: Chromosomal aberrations
NCA: Non-chromosomal aberrations
PRISMA: Preferred Reporting Items for Systematic Reviews and Meta-Analyses
ej-ICSI: ejaculated sperm ICSI
TESE: Testicular sperm extraction
IVM: In vitro maturation
